# Genome-Wide Association Studies for Five Forage Quality-Related Traits in Sorghum (*Sorghum bicolor* L.)

**DOI:** 10.3389/fpls.2018.01146

**Published:** 2018-08-21

**Authors:** Jieqin Li, Weijie Tang, Ya-Wen Zhang, Kai-Ning Chen, Chenchen Wang, Yanlong Liu, Qiuwen Zhan, Chunming Wang, Shi-Bo Wang, Shang-Qian Xie, Lihua Wang

**Affiliations:** ^1^College of Agriculture, Anhui Science and Technology University, Fengyang, China; ^2^College of Horticulture, Institute of Tropical Agriculture and Forestry, Hainan University, Haikou, China; ^3^National Key Laboratory of Crop Genetics and Germplasm Enhancement, Jiangsu Plant Gene Engineering Research Center, Nanjing Agricultural University, Nanjing, China; ^4^College of Plant Science and Technology, Huazhong Agricultural University, Wuhan, China

**Keywords:** sorghum, GWAS, forage quality-related traits, mrMLM, QTNs

## Abstract

Understanding the genetic function of the forage quality-related traits, including crude protein (CP), neutral detergent fiber (NDF), acid detergent fiber (ADF), hemicellulose (HC), and cellulose (CL) contents, is essential for the identification of forage quality genes and selection of effective molecular markers in sorghum. In this study, we genotyped 245 sorghum accessions by 85,585 single-nucleotide polymorphisms (SNPs) and obtained the phenotypic data from four environments. The SNPs and phenotypic data were applied to multi-locus genome-wide association studies (GWAS) with the mrMLM software. A total of 42 SNPs were identified to be associated with the five forage quality-related traits. Moreover, three and two quantitative trait nucleotides (QTNs) were simultaneously detected among them by three and two multi-locus methods, respectively. One QTN on chromosome 5 was found to be associated simultaneously with CP, NDF, and ADF. Furthermore, 3, 2, 2, 5, and 2 candidate genes were identified to be responsible for CP, NDF, ADF, HC, and CL contents, respectively. These results provided insightful information of the forage quality-related traits and would facilitate the genetic improvement of sorghum forage quality in the future.

## Introduction

Sorghum (*Sorghum bicolor* L.) is a popular crop worldwide, which is used a food source, animal fodder, and raw material for alcoholic beverages and biofuels in industries (Paterson et al., 2009). Most of the important agronomic traits are genetically controlled by quantitative trait loci (QTLs) (Zou et al., 2012; Boyles et al., 2017). For example, the forage quality is an important quantitative trait. Thus, understanding their genetic mechanism is essential for identifying the candidate genes and selecting effective molecular markers in sorghum breeding.

The forage digestibility and crude protein (CP) content are the main focus for forage sorghum breeding (Murray et al., 2008). Forage digestibility is mainly determined by the cellulose (CL), hemicellulose (HC), and lignin contents (Wang H. et al., 2016), which are important components of the neutral detergent fiber (NDF). On the other hand, acid detergent fiber (ADF) is a portion of sorghum fiber and is obtained from acid detergent-treated forage. The two types of fibers, NDF and ADF, are the two vital components of forage digestibility. Recently, the forage quality traits have been studied in sorghum and some related QTLs have been identified (Murray et al., 2008; Shiringani and Friedt, 2011; Li et al., 2015). However, these identified QTLs were observed to be less sensitive due to the limitation of linkage analysis based on bi-parental mapping populations.

Compared with the linkage analysis of bi-parental mapping populations, genome-wide association studies (GWAS), which is based on linkage disequilibrium (LD) and provided sufficient genetic background information, have become a powerful alternative for the investigation of quantitative traits. There are three main strategies for GWAS. Firstly, a generalized linear model (GLM) was proposed for the genetic analysis of the quantitative traits (Price et al., 2006), but it did not effectively control the polygenic background. Secondly, a mixed linear model (MLM) was elaborated to take into account the population structure and polygenic background using the pedigree relationship or marker information (Zhang et al., 2005; Yu et al., 2006). These methods involve a large calculation burden due to the tremendous number of existing markers. Therefore, a series of rapid detection methods were finally developed, such as EMMA (Kang et al., 2008), FaST-LMM (Lippert et al., 2011), GRAMMAR-Gamma (Svishcheva et al., 2012), ECMLM (Li et al., 2014), SUPER (Wang et al., 2014), BOLT-LMM (Loh et al., 2015), and FarmCPU (Liu et al., 2016). Although the above methods have been widely adopted, the complex traits controlled by multiple QTNs could not be effectively identified. To address this issue, Zhang's group has developed a series of multi-locus GWAS methods, including mrMLM (Wang S. B. et al., 2016), FASTmrMLM (Tamba et al., 2017), FASTmrEMMA (Wen et al., 2017), ISIS EM-BLASSO (Tamba et al., 2017), pLARmEB (Zhang et al., 2017), and pKWmEB (Ren et al., 2018).

In our study, we utilized the advantageous multi-locus GWAS to investigate the sorghum forage quality-related traits. We genotyped 245 sorghum accessions by using 85,585 single-nucleotide polymorphisms (SNPs) and phenotyped them in the four environments. The data were analyzed by the multi-locus GWAS software, mrMLM.

## Materials and methods

### Plant materials

The 245 sorghum accessions (Table S1) included 238 mini-core collection sorghum and 7 breeding varieties. These accessions were planted in the Fengyang campus of Anhui Science and Technology University (Fengyang, China, 32°52′ N, 177°33′ E) and Tengqiao town of Hainan Province (Tengqiao, China, 18°24′ N, 109°45′ E) in 2015 and 2016. All the experiments in the four environments used a completely randomized block design with three replicates. The aboveground parts were harvested when 70% accessions were at the heading stage. The harvested plants were dried at 75°C for three days. The plant material was then milled using a grinder and filtered using a 0.5 mm sieve.

### Phenotypic trait evaluation and data analysis

Seven hundred and thirty-five sorghum samples (3 replicates) were measured for CP, CL, HC, NDF, and ADF using the traditional chemical methods, and simultaneously scanned for near-infrared (NIR) spectra with an Antaris™ II FT-NIR Analyzer (Thermo, USA). A model was established using TQ Analyst software based on the NIR spectra and the results of the chemical analysis. The samples were then scanned for NIR spectra, and their CP, CL, HC, NDF, and ADF were calculated using the model. The mean of the phenotypic data and the correlation coefficients were calculated using Microsoft Excel.

### DNA extraction and RAD sequencing

Total DNA was extracted using the DNAsecure Plant Kit (Qiagen, Cat.No. DP320). All the samples were standardized to 50 ng/μL, and 10 μL of each sample was digested with the enzymes, PstI (CTGCAG) and MspI (CCGG), at 37°C for 2 h and then at 65°C for 20 min. The digested samples were ligated with the adapters from Illumina (San Diego, CA, USA). The ligated samples were then pooled using the same volume (10 μL) for PCR-amplification in a single tube. The fragment length was analyzed using a Bioanalyzer (Agilent), and the PCR products were quantified by a Qubit3.0 fluorometer (Invitrogen). The GBS library was run on an Illumina Hiseq2500 (San Diego, CA, USA).

### RAD-seq data and population structure analysis

The sequencing reads of the 245 samples were extracted from the raw data of RAD-seq and filtered by using fastx_barcode_splitter and fastq_quality_filter with parameters (-q 20 -p 80 -Q 33) of fastx_toolkit-0.0.13.2 (http://hannonlab.cshl.edu/fastx_toolkit/). The high-quality sequencing data were aligned using BWA MEM (Li and Durbin, 2009). The software—samtools, mpileup, and bcftools (Li et al., 2009), were then used to call the SNPs from the alignment files of the 245 samples; these were kept as the genotype of the sorghum population. These genotypic data were used to calculate the population structure using the fastSTRUCTURE software (Raj et al., 2014).

### Genome-wide association studies

The GWAS for the five forage quality-related traits (CP, CL, HC, NDF, and ADF) was performed using six methods, including mrMLM, FASTmrMLM, FASTmrEMMA, pLARmEB, pKWmEB, and ISIS EM-BLASSO in the mrMLM software. The main model used in this study in the mrMLM software is as follows :*y* = *Wα*+*Xβ*+*Zu*+ε, where *y* is an *n* × 1 phenotypic vector of quantitative traits, and *n* is the number of accessions. *W* = (ω_1_, ω_2_, ⋯ , ω_*c*_) is an *n* × *c* matrix of covariates (fixed effects), including a column vector of 1; the population structure or principal components can be incorporated into _*W*_. Moving on, α is a *c* × 1 vector of fixed effects, including the intercept, and *X* is an *n* × 1 vector of marker genotypes. $\beta ∽N\left(0,\sigma_{\beta }^{2}\right)$is the random effect of putative QTN. *Z* is an *n* × *m* design matrix, and $u∽MVN_{m}\left(0,\sigma_{g}^{2}K\right)$ is an *m* × 1 vector of polygenic effects. *K* is a known *n* × *n* relatedness matrix. $\varepsilon ∽MVN\left(0,\sigma_{e}^{2}I_{n}\right)$ is an *n* × 1 vector of residual errors, and $\sigma_{e}^{2}$ is residual variance. *I*_*n*_ is an *n* × *n* identity matrix, and MVN denotes multivariate normal distribution. An LOD score of 3 was used as the critical threshold for significant QTNs for all the six methods.

### Identification of candidate genes

Genes that were hit directly by the associated QTNs within a 50-kb stretch were selected to choose the candidate genes as described in Upadhyaya et al. (2016). The physical locations of the QTNs were recorded according to the assembly genome (Sorghum_bicolor_NCBIv3) and the annotation GFF file (https://www.ncbi.nlm.nih.gov/genome/108). The detailed functions of the corresponding genes were annotated by performing BLASTP search at the NCBI website, and the candidate genes were assigned to different biological processes based on the function of their homologs in other species in literature or with the help of data in the Conserved Domains Database. The selected candidate genes were associated with the main QTNs of the five traits if they made a contribution (*r*^2^) greater than 5%.

## Results

### Phenotype analysis

Extensive phenotypic variations of CP, CL, HC, NDF, and ADF were observed in the 245 sorghum samples in the four environments, including two locations in 2 years (Fengyang and Tengqiao in 2015 and 2016, Table 1). The variation range of the five traits was 1.5 to 3.5-fold: the phenotype values of the CP content were 3.80 to 13.24% with 2.5 to 3.5-fold variation. The NDF content varied from 0.38 to 0.75 g/g with 1.5 to 1.9-fold variation, while the ADF content varied from 0.18 to 0.52 g/g with a 1.8 to 2.2-fold variation. Lastly, the HC and CL contents varied from 0.14 to 0.42 g/g and 0.12 to 0.45 g/g with 1.6 to 2.2-fold and 1.8 to 2.8-fold variations, respectively.

**Table 1:** The statistical description for CP, CL, HC, NDF, and ADF in 245 sorghum accessions in the four environments.

**Trait-environment**	**Mean**	**Range**	**SD**	**CV (%)**
CP-2015fy	5.9005	3.25–11.25	0.950	16.10
CP-2015hn	8.5280	4.82–12.86	0.892	10.46
CP-2016fy	6.1888	3.80–10.41	0.640	10.34
CP-2016hn	8.7070	5.24–13.24	0.953	10.95
CL-2015fy	0.3219	0.1993–0.4504	0.0463	14.38
CL-2015hn	0.2787	0.1632–0.3914	0.0374	13.42
CL-2016fy	0.3115	0.2125–0.3851	0.0285	9.15
CL-2016hn	0.2661	0.1210–0.3453	0.0351	13.19
HC-2015fy	0.2592	0.1951–0.4165	0.0271	10.46
HC-2015hn	0.2653	0.1501–0.3298	0.0277	10.44
HC-2016fy	0.2365	0.1816–0.2992	0.0219	9.26
HC-2016hn	0.2613	0.1437–0.3185	0.0238	9.11
NDF-2015fy	0.6463	0.4327–0.7513	0.0692	10.71
NDF-2015hn	0.5999	0.3839–0.7414	0.0576	9.60
NDF-2016fy	0.6115	0.4734–0.7198	0.0434	7.10
NDF-2016hn	0.6024	0.4431–0.7282	0.0716	11.89
ADF-2015fy	0.3870	0.2376–0.5208	0.5340	13.80
ADF-2015hn	0.3341	0.2148–0.4687	0.0433	12.96
ADF-2016fy	0.3750	0.2501–0.4504	0.0382	10.19
ADF-2016hn	0.3124	0.1817–0.4096	0.0355	11.36

*The unit of CP is % and that of the other four traits is g/g. Two locations: Fengyang (fy) and Tengqiao (hn); 2 years: 2015 and 2016*.

The correlation coefficients between a pair of traits were assessed. It was revealed that there were significant and positive correlations between ADF, NDF, CL, and HC. However, they correlated significantly but negatively with the CP phenotype, except for HC in 2015fy, 2015hn, and 2016fy and NDF in the 2016fy environments (Table S2). These results indicated that the four traits of ADF, NDF, HC, and CL could be genetically linked or that some genes could play pleiotropic roles in controlling these phenotypes.

### RAD-seq genotyping and population structure

**Figure 1:**
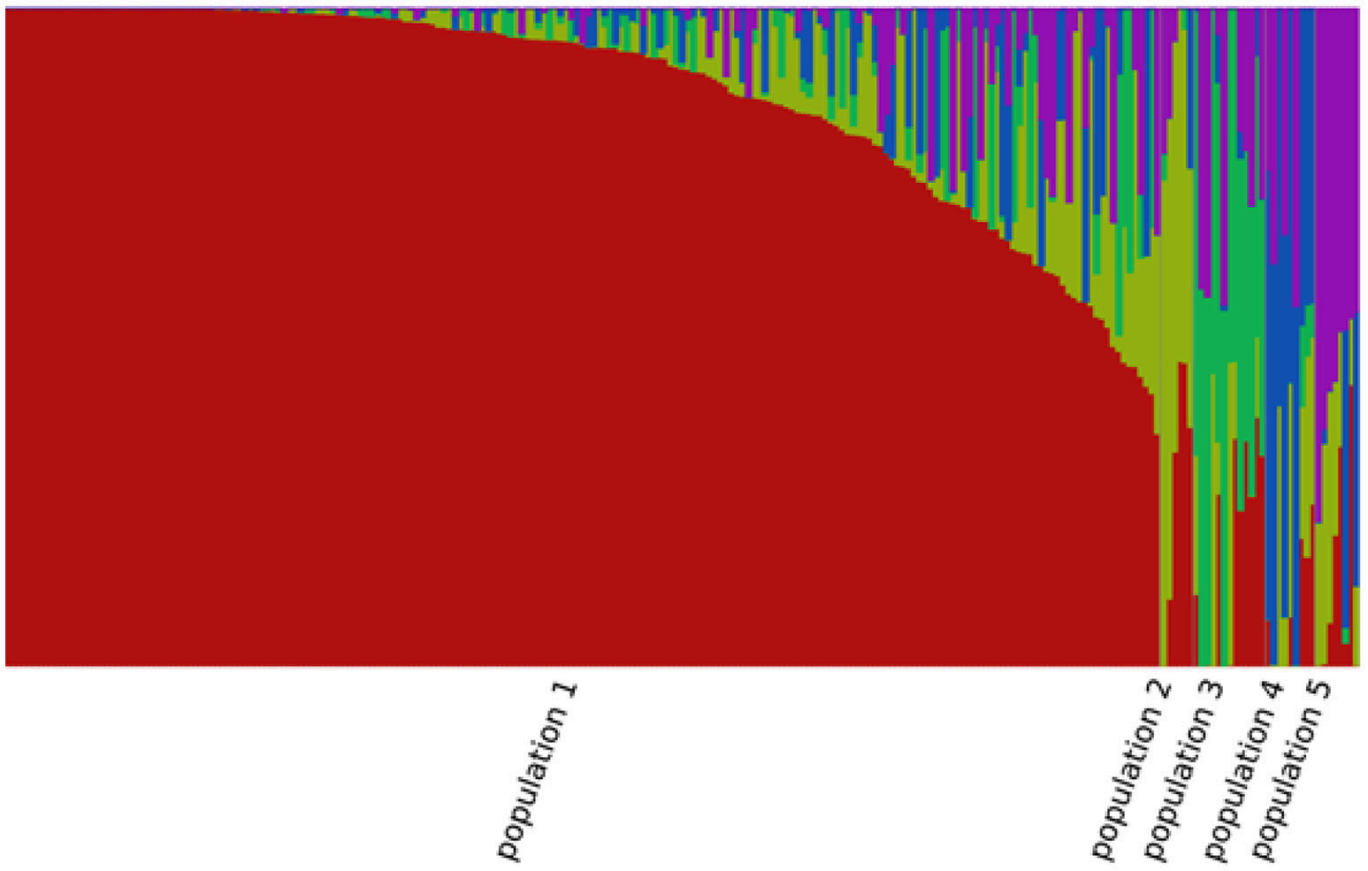
Population structure of the 245 sorghum accessions.

A total of 85,585 SNPs were identified in the genotypes of the 245 accessions using RAD-seq (Table 2). Chromosome 1 had the most SNP markers (11,719), while chromosome 10 had the least (5,994). The highest SNP density was observed on chromosome 3 with 1.5 SNP markers per 10 kb, whereas the lowest density was on chromosome 7 with 0.9 SNP markers per 10 kb. The average density was 1.2 markers per 10 kb. Altogether, the genotyping results were of high quality in this research. The population structure was analyzed using the fastSTRUCTURE software. The results showed that the best value for the number of sub-populations was 5 (Figure 1), which was selected to perform further GWAS analysis.

**Table 2:** Number of SNPs on the 10 chromosomes of sorghum.

**Chromosome**	**Length (kb)**	**No. of SNPs**	**SNP density (SNP/10 kb)**
1	80884.392	11,719	1.4
2	77742.459	11,040	1.4
3	74386.277	11,181	1.5
4	68658.214	8,900	1.3
5	71854.699	7,958	1.1
6	61277.060	8,266	1.3
7	65505.356	6,086	0.9
8	62686.529	6,731	1.1
9	59416.394	7,710	1.3
10	61233.695	5,994	1

### GWAS using six multi-locus methods

Six methods in the mrMLM software were used for the detection of QTNs. A total of 42 significant QTNs were detected for the five forage quality-related traits (CP, CL, HC, NDF, and ADF) across the four environments using six methods (Table 3). There were 5, 3, 3, 24, and 7 QTNs that were associated with CP, CL, HC, NDF, and ADF, respectively. Each trait was controlled by multiple QTNs. The 5 SNPs associated with the CP content were identified on chromosomes 2, 5, 7, and 9. The 3 SNPs associated with the CL content were present on chromosomes 2, 5, and 8, while the 3 SNPs associated with the HC content were located on chromosomes 1 and 9. The 24 SNPs associated with NDF were present on chromosomes 1, 2, 6, 7, 8, 9, and 10. Lastly, the 7 SNPs associated with the ADF content were present on chromosomes 3, 4, 5, 8, and 10. Among these QTNs, there were 4 significant QTNs, each of which was responsive for more than one trait. The three traits of ADF, CL, and NDF were associated with one QTN on chromosome 5 (RSS50197); both CL and NDF were associated with two QTNs (RSS21890 and RSS76122); ADF and CL were associated with one QTN (RSS68908) on chromosome 8.

**Table 3:** QTNs for CP, CL, HC, NDF, and ADF in the four environments using six multi-locus GWAS methods.

**Trait**	**QTN**	**Chr**	**Pos (bp)**	**Environment**	**GWAS**			
					**Method**	**Effect**	**LOD**	***r*^2^ (%)**
CP	RSS17673	2	60877961	2016hn	pKWmEB pLARmEB	−0.7575 −0.0014	5.11 5.11	10.89 2.87E-05
	RSS22092	2	74893615	2015fy	pKWmEB pLARmEB	−0.9453 −0.0014	4.17 3.31	9.37 1.28E-05
	RSS48493	5	65128246	2016hn	FASTmrEMMA	3.0389	4.56	5.82
	RSS62628	7	56446694	2016hn	EM_BLASSO	−0.8022	3.95	4.53
	RSS72060	9	564198	2015hn	pLARmEB	0.002	3.63	3.61E-05
CL	RSS21890	2	74389054	2015hn	FASTmrMLM EM_BLASSO pKWmEB	0.0259 0.026 0.026	3.99 4.49 4.75	10.58 10.62 14.85
	RSS50197	5	70141003	2015fy	FASTmrMLM pKWmEB	3.05E-05 3.45E-05	3.17 3.17	5.77E-06 6.08
	RSS68908	8	52710244	2016hn	pLARmEB	−0.0017	3.47	1.42E-02
HC	RSS1510	1	7334364	2015hn	FASTmrMLM EM_BLASSO pKWmEB	0.0273 0.0265 0.0265	5.32 5.36 5.36	7.70 7.27 10.37
	RSS3431	1	16641334	2016hn	EM_BLASSO	−2.06E-05	3.14	3.55E-06
	RSS76122	9	48549474	2016hn	FASTmrMLM pKWmEB	0.025 0.026	4.93 3.33	5.24 7.74
NDF	RSS973	1	5021420	2015fy	pLARmEB	−0.0011	9.61	9.00E-04
	RSS2859	1	12968078	2015hn	pLARmEB	0.0031	4.49	1.47E-02
	RSS3945	1	19028439	2015hn	pLARmEB	−0.001	5.26	6.00E-04
	RSS4300	1	21729035	2015fy	FASTmrMLM	−5.22E-05	3.91	5.72E-06
	RSS7796	1	66643031	2015hn	EM_BLASSO	0.0435	4.12	10.89
	RSS14238	2	9529690	2015hn	pLARmEB	0.0011	4.50	1.17E-03
	RSS21890	2	74389054	2015hn	pKWmEB	0.0419	4.80	15.72
	RSS44026	5	4665028	2015fy	FASTmrMLM	−1.00E-04	3.70	2.00E-04
	RSS50197	5	70141003	2015fy	pKWmEB	0.0428	4.58	7.07
	RSS54219	6	47408694	2015hn	pLARmEB	0.0014	3.37	1.58E-03
	RSS55031	6	49852623	2015hn	pLARmEB	−0.0013	4.35	1.24E-03
	RSS58234	6	58636709	2015fy	pLARmEB	0.002	3.80	1.89E-03
	RSS61032	7	8098766	2015hn	pLARmEB	0.0019	4.07	2.19E-03
	RSS65142	7	65417969	2015hn	pLARmEB	0.003	3.17	7.65E-03
	RSS65800	8	2362643	2015hn	pLARmEB	0.0012	3.64	1.15E-03
	RSS65801	8	2362646	2015hn	pLARmEB	0.0011	3.77	8.00E-04
	RSS66600	8	5538354	2015hn	pLARmEB	0.0017	3.28	2.12E-03
	RSS68217	8	48827265	2015hn	pLARmEB	0.0016	3.90	2.12E-03
	RSS70856	8	60604340	2015fy	pKWmEB	0.044	4.08	7.77
	RSS72128	9	886296	2015hn	pLARmEB	0.0013	3.46	5.00E-04
	RSS72803	9	2831721	2015fy	pLARmEB	0.002	3.61	4.72E-03
	RSS76122	9	48549474	2016hn	pKWmEB	0.0302	3.87	5.99
	RSS79370	9	58586399	2015fy	pLARmEB	−0.0037	3.76	1.33E-02
	RSS81889	10	12257429	2015hn	pLARmEB	−0.0012	3.15	2.21E-03
ADF	RSS29915	3	60813170	2015fy	mrMLM	0.0763	4.41	20.24
	RSS35476	4	6415156	2015hn	EM_BLASSO pKWmEB	−0.0159 −9.00E-04	3.27 3.38	2.98 4.43
	RSS40375	4	60973382	2015hn	EM_BLASSO	0.0144	3.51	3.09
	RSS50197	5	70141003	2015fy	FASTmrMLM EM_BLASSO pLARmEB	0.0292 0.0292 0.0032	4.42 4.42 3.64	3.97 3.97 4.91E-02
	RSS68908	8	52710244	2016hn	pLARmEB	−0.0018	3.44	1.41E-02
	RSS79627	10	386174	2015fy	pLARmEB	0.0047	3.10	0.28
	RSS83457	10	50561994	2015hn	FASTmrMLM pKWmEB	0.0178 0.0175	3.15 3.26	4.25 7.09

Among the above six methods, pLARmEB was the most powerful and accountable for the identification of the 24 QTNs that mainly contributed to the NDF content trait (17 QTNs); however, their contributions were less than what were detected by other methods, except for one major QTN (RSS17673), whose contribution was greater than 5% (Table 3). The other methods of PKWmEB, ISIS EM-BLASSO, FASTmrMLM, mrMLM, and FASTmrEMA identified 12, 8, 8, 1, and 1 QTNs, respectively. About 43% (13 of 30) of these SNPs included the major QTNs (*r*^2^ > 5%). Besides, 3 QTNs (RSS50197, RSS21890, and RSS1510) were detected simultaneously by 3 methods, and another 5 QTNs (RSS35476, RSS83457, RSS76122, RSS22092, and RSS17673) were identified simultaneously by 2 methods. The remaining QTNs were detected by a single method, but most of them were considered as reliable because of the high thresholds at which they were detected.

### Identification of candidate genes

The assembled sorghum genome and the annotation file from NCBI were used to annotate the genes associated with the significant QTNs. There were 14 candidate genes for five forage quality-related traits. The NDF and CP content traits were associated with five and three candidate genes, respectively. The remaining 6 genes were related to the CL, HC, and ADF content traits with each trait being associated with two genes (Table 4).

**Table 4:** Candidate genes for CP, CL, HC, NDF, and ADF traits.

**Trait**	**Chr**.	**QTNs**	**Start**	**End**	**Gene**	**Function**
CP	2	RSS17673	60921089	60923833	Sobic.002G217100	Serine/threonine-protein kinase
	2	RSS22092	74887469	74892115	Sobic.002G397001	Cysteine proteinase
	5	RSS48493	65140867	65142310	Sobic.005G171700	Uncharacterized protein
CL	2	RSS21890	74372243	74384041	Sobic.002G390800	Kinesin-like protein
	5	RSS50197	70130797	70134481	Sobic.005G215300	Laccase-15
HC	1	RSS1510	7364358	7367682	Sobic.001G095700	Transcription factor bHLH
	9	RSS76122	48547319	48550332	Sobic.009G132000	Uncharacterized protein
NDF	1	RSS7796	66642749	66650461	Sobic.001G378300	Sucrose synthase
	2	RSS21890	74372243	74384041	Sobic.002G390800	Kinesin-like protein
	5	RSS50197	70130797	70134481	Sobic.005G215300	Laccase-15
	8	RSS70856	60570342	60574129	Sobic.008G172200	Transcription factor TCP
	9	RSS76122	48547319	48550332	Sobic.009G132000	Uncharacterized protein
ADF	3	RSS29915	60842731	60844801	Sobic.003G272200	Transcription factor bHLH
	10	RSS83457	50554160	50558010	Sobic.010G172100	Transcription factor bHLH

For the CP content trait, one candidate gene that was associated with the major QTN (RSS17673) encoded a serine/threonine-protein kinase (Sobic.002G217100), which was consistent with a previous study that concluded that serine/threonine-protein kinases are involved in signal cascade for nitrogen metabolism in plants (Champigny, 1995). Besides, two candidate genes were identified for the CP content on chromosomes 2 and 5 with one gene encoding a cysteine proteinase and the other encoding an uncharacterized protein. In addition, one main QTN associated with the CL content trait on chromosome 2 was identified, and the associated candidate gene encoded a kinesin-like protein. The kinesin protein is reported to be involved in the deposition of CL during secondary growth of fiber cells in Arabidopsis (Kong et al., 2015). Furthermore, 5 main QTNs were detected in association with the NDF content; two of these (RSS21890 and RSS50197) were co-localized with those for the CL content trait. Therefore, the same two candidate genes were identified for the NDF and CL content (Sobic.005G215300 and Sobic.002G390800). For the ADF content trait, 2 main QTNs were detected on chromosomes 3 and 10, where both candidate genes encoded a bHLH transcription factor (Sobic.003G272200 and Sobic.010G172100).

## Discussion

Genome-wide association study is an important alternative for mapping quantitative traits. It has been applied rapidly and extensively in plant research. These methods have been widely adopted, but only a few QTNs for each complex trait have been identified. In this study, we implemented the latest multi-locus GWAS methods available in mrMLM (Wang S. B. et al., 2016; Tamba et al., 2017; Wen et al., 2017; Zhang et al., 2017; Ren et al., 2018), which can effectively overcome the above issue and actively detect the QTNs associated with the quantitative traits. Six methods in the mrMLM software were used to identify the QTNs of five forage quality-related traits in sorghum. Of these methods, pLARmEB detected the most significant QTNs, but most of them contributed insignificantly to heritability (Table 3). Most of the significant QTNs associated with the NDF content, detected using pLARmEB, were observed to be in the 2015hn (13 QTNs) and 2015fy (4 QTNs) environments (Table 3). This result might be associated with the range of values for this phenotypic trait (Table 1) and the difference of environments between Hainan Tengqiao (18°24′ N, 109°45′ E) and Anhui Fengyang (32°52′ N, 177°33′ E). The range of NDF-2015hn and NDF-2015fy was 0.36 and 0.32, which was higher than that in 2016hn (0.28) and 2016fy (0.25), respectively (Table 1). Similar conclusions can be drawn for other traits. It means that the greater the difference in phenotype, the more favorable it is for the detection of the associated QTNs. Hainan and Anhui are located in the tropics and subtropics, respectively, where the environment is particularly different in different climatic zones. The previous study has revealed that the climatic conditions, including temperature, water availability, and soil, are important factors which affect the forage quality of sorghum (Hussin et al., 2007). In our study, the QTN RSS50197 associated with the ADF, CL, and NDF traits was uniquely detected in the same environment of 2015fy by using three GWAS methods. The above results revealed the influence of environment in QTN detection. However, the latest methods of multi-locus GWAS applied in our study are currently unable to detect the QTN-by-environment interaction. Thus, we hope that in the future new methods can be developed by the theoretical researchers.

According to the GWAS analysis, 5, 3, 3, 7, and 24 QTNs were identified for CP, CL, HC, ADF, and NDF content, respectively. Of the 5 candidate loci for the CP content, 2 were already identified in the previous studies. The locus on chromosome 9 was mapped in the same region by Murray et al. (2008) and Li et al. (2015) in sorghum as well. Of the 3 candidate loci for the CL content, 2 were identified in the same region on chromosomes 2 and 8 by Murray et al. (2008) and Shiringani and Friedt (2011). Similarly, of the 7 loci for the ADF content, 2 were mapped on chromosome 4, which was in agreement with the report of Shiringani and Friedt (2011). As for the 24 loci for the NDF content, the 2 loci on chromosome 6 and 1 loci on chromosome 8 were also identified by Shiringani and Friedt (2011). More importantly, several QTNs that were detected by the six methods in this study were novel identifications for forage quality-related traits in sorghum.

The QTLs for the NDF or ADF content co-localized with those for the CL or HC content, which has been reported previously in sorghum. Cardinal et al. (2003) reported colocalization of QTLs that are associated with the cell wall components, such as lignin, NDF, and ADF in stalks of maize. Murray et al. (2008) and Shiringani and Friedt (2011) also found colocalization of QTLs associated with the CL, HC, NDF, and ADF content traits in sorghum by QTL mapping. In this study, we detected 4 co-localized QTNs: 1 for three traits and 3 for two traits. All of these QTNs were associated with NDF or ADF and with CL or HC. NDF is mainly composed of CL, HC, and lignin, while ADF is composed of CL and lignin. The difference between NDF and ADF is whether they have HC as a component or not. Furthermore, we found that NDF and ADF significantly correlated with CL or HC. It is reasonable that these QTNs were co-localized.

Both NDF and ADF include CL and lignin. There are a series of reports about the biosynthesis and signaling pathways of CL and lignin in plants (Kim et al., 2013; McNamara et al., 2015; Yoon et al., 2015; Chezem and Clay, 2016). In this study, we identified 5 and 2 candidate genes for the NDF and ADF content traits, respectively. Of these candidate genes, 1 gene (Sobic.001G378300) encoded a sucrose synthase, which is an integral component of the CL synthesis mechanism. Gerber et al. (2014) reported that deficient sucrose synthase activity in developing wood does not specifically affect the CL biosynthesis but causes an overall decrease in the cell wall polymers. Furthermore, Poovaiah et al. (2014) reported that the lignin content increases in all the transgenic switchgrass lines, where sucrose synthase (PvSUS1) was overexpressed.

Lignin, CL, and HC are the main components of secondary cell walls (Zhong et al., 2011). Secondary cell wall biosynthesis is positively regulated by NAD and MYB transcription factors (Zhong and Ye, 2014; Chezem and Clay, 2016). Moreover, studies have also identified several transcription factors (e.g., WRKY, ERF, and bHLH) that regulate the biosynthesis of secondary walls (Kim et al., 2013; Taylor-Teeples et al., 2015; Chezem and Clay, 2016). In this study, we identified a candidate gene encoding a bHLH transcription factor for CL and two bHLH genes for ADF. These transcription factors might also be involved in the regulation of CL or lignin biosynthesis. The function of the candidate genes identified in this work needs to be studied further by transformation experiments in the future.

## Author contributions

JL, LW, and S-QX designed and conceived the experiments. Y-WZ, K-NC, and S-BW performed the computational analysis. WT and JL extracted the DNA and performed the experimental analysis. CCW and YL assisted with experiments in data collection and analysis. QZ and CW participated in the design and supervised the study. S-QX and JL discussed the results and interpretation of the final data. S-QX and JL drafted the manuscript. All authors read and approved the final manuscript.

### Conflict of interest statement

The reviewer ML declared a shared affiliation, though no other collaboration, with several of the authors WT, CW to the handling Editor. The remaining authors declare that the research was conducted in the absence of any commercial or financial relationships that could be construed as a potential conflict of interest.
