# Supplementary material for: Genome-Wide Association Studies for Five Forage Quality-Related Traits in Sorghum (Sorghum bicolor L.)
Source: Front Plant Sci. 2018 Aug 21;9:1146. doi: 10.3389/fpls.2018.01146 (PMC6111974; doi:10.3389/fpls.2018.01146)
Supplement: Supplementary file 2 [file Table_2.DOCX]

Table S2. Correlation coefficients among 5 traits in four environments (2015fy/2015hn/2016fy/2016hn)

|  | ADF | CL | HC | NDF | CP |
| --- | --- | --- | --- | --- | --- |
| ADF | 1 |  |  |  |  |
| CL | 0.95**/0.98**/0.81**/0.98** | 1 |  |  |  |
| HC | 0.42**/0.32**/0.34**/0.65** | 0.43**/0.41**/0.52**/0.68** | 1 |  |  |
| NDF | 0.93**/0.89**/0.86**/0.86** | 0.90**/0.92**/0.97**/0.88** | 0.71**/0.71**/0.47**/0.94** | 1 |  |
| CP | -0.39**/-0.66**/-0.24**/-0.47** | -0.29**/-0.66**/-0.18**/-0.46* | -0.07/-0.07/-0.13/-0.53** | -0.26*/-0.57*/-0.13/-0.53* | 1 |

** Means significantly different at 0.01 level
